# Comparison of CSF markers and semi-quantitative amyloid PET in Alzheimer’s disease diagnosis and in cognitive impairment prognosis using the ADNI-2 database

**DOI:** 10.1186/s13195-017-0260-z

**Published:** 2017-04-26

**Authors:** Fayçal Ben Bouallègue, Denis Mariano-Goulart, Pierre Payoux, the Alzheimer’s Disease Neuroimaging Initiative (ADNI)

**Affiliations:** 1 Toulouse NeuroImaging Centre (ToNIC), Université de Toulouse, Inserm/UPS, Toulouse, France; 2 Nuclear Medicine Department, Purpan University Hospital, Toulouse, France; 3 Nuclear Medicine Department, Lapeyronie University Hospital, Montpellier, France

**Keywords:** Alzheimer’s disease, MCI, Amyloid PET, CSF markers, ADNI

## Abstract

**Background:**

The relative performance of semi-quantitative amyloid positron emission tomography (PET) and cerebrospinal fluid (CSF) markers in diagnosing Alzheimer’s disease (AD) and predicting the cognitive evolution of patients with mild cognitive impairment (MCI) is still debated.

**Methods:**

Subjects from the Alzheimer’s Disease Neuroimaging Initiative 2 with complete baseline cognitive assessment (Mini Mental State Examination, Clinical Dementia Rating [CDR] and Alzheimer’s Disease Assessment Scale–Cognitive Subscale [ADAS-cog] scores), CSF collection (amyloid-β_1–42_ [Aβ], tau and phosphorylated tau) and ^18^F-florbetapir scans were included in our cross-sectional cohort. Among these, patients with MCI or substantial memory complaints constituted our longitudinal cohort and were followed for 30 ± 16 months. PET amyloid deposition was quantified using relative retention indices (standardised uptake value ratio [SUVr]) with respect to pontine, cerebellar and composite reference regions. Diagnostic and prognostic performance based on PET and CSF was evaluated using ROC analysis, multivariate linear regression and survival analysis with the Cox proportional hazards model.

**Results:**

The cross-sectional study included 677 participants and revealed that pontine and composite SUVr values were better classifiers (AUC 0.88, diagnostic accuracy 85%) than CSF markers (AUC 0.83 and 0.85, accuracy 80% and 75%, for Aβ and tau, respectively). SUVr was a strong independent determinant of cognition in multivariate regression, whereas Aβ was not; tau was also a determinant, but to a lesser degree. Among the 396 patients from the longitudinal study, 82 (21%) converted to AD within 22 ± 13 months. Optimal SUVr thresholds to differentiate AD converters were quite similar to those of the cross-sectional study. Composite SUVr was the best AD classifier (AUC 0.86, sensitivity 88%, specificity 81%). In multivariate regression, baseline cognition (CDR and ADAS-cog) was the main predictor of subsequent cognitive decline. Pontine and composite SUVr were moderate but independent predictors of final status and CDR/ADAS-cog progression rate, whereas baseline CSF markers had a marginal influence. The adjusted HRs for AD conversion were 3.8 (*p* = 0.01) for PET profile, 1.2 (*p* = ns) for Aβ profile and 1.8 (*p* = 0.03) for tau profile.

**Conclusions:**

Semi-quantitative amyloid PET appears more powerful than CSF markers for AD grading and MCI prognosis in terms of cognitive decline and AD conversion.

## Background

Mild cognitive impairment (MCI) refers to cognitive deficits that do not directly impact the activities of daily living [1] and may be related to varied aetiologies, including depression, dementia and cerebrovascular disease. Only a small proportion of patients with MCI will convert to Alzheimer’s disease (AD) within a given period of time, whereas the others will incur a variable cognitive decline or even revert to normal [2]. Considerable effort has been devoted to identifying and developing reliable biomarkers of incipient AD to target the individuals who would most benefit from early treatment intervention [3]. Decreased cerebrospinal fluid (CSF) concentration of the amyloid-β_1–42_ peptide (Aβ) and an increased level of the protein tau are seen in patients with AD [4, 5]. This pathological CSF signature is a key feature in AD diagnosis, and the CSF profile, potentially combined with neuroimaging data [6–9], has the ability to predict cognitive decline and conversion to AD independently of established risk factors such as age, sex and apolipoprotein E (ApoE) genotype [10–13].

Positron emission tomography (PET) using ^11^C-labelled Pittsburgh Compound B (PiB) or fluorinated tracers such as ^18^F-florbetapir allows in vivo visualisation and quantification of cortical Aβ deposition with high sensitivity and specificity compared with amyloid plaque burden at autopsy [14, 15]. Therefore, amyloid PET was included as a pathophysiological marker in the most recent international working group diagnostic criteria [16]. Although standard interpretation relies on visual assessment, semi-quantitative measures of cortical retention with respect to a reference subcortical region is expected to provide refined evaluation of the amyloid burden with high test-retest reliability [17]. Historically, normalisation of standardised uptake value (SUV) has been done using the brainstem, pons or whole cerebellum as the reference region. However, there is growing evidence that composite reference regions that include some subcortical white matter induce less temporal variability in sequential measurements, yielding higher accuracy in assessing subtle time changes and greater power to detect Aβ accumulation [18–20]. Researchers in several studies have reported the capacity of amyloid PET using fluorinated tracers (either visual [21], semi-quantitative [22, 23] or both [24]) to provide prognostic insight regarding cognitive decline and conversion to AD in patients with MCI, in line with previous evidence of the prognostic value of PiB PET [25–29]. A recent multi-centre study demonstrated the clinical impact of florbetapir PET in terms of diagnostic confidence and drug treatment [30].

Although CSF and PET measures of Aβ deposition are highly correlated [31–34], the comparative relevance of these two markers in discriminating patients with AD and predicting cognitive outcome in patients with MCI is still under debate [35]. Hake et al. showed that CSF and PET profiles were both discriminant in classifying healthy control subjects and patients with MCI vs patients with AD [36]. Palqvist et al. found that the PET standardised uptake value ratio (SUVr) was associated with disease stage (cognition, memory and hippocampal volume) in patients with MCI, whereas CSF markers were not [37]. In recent studies, researchers concluded that CSF analysis might detect Aβ deposition earlier than PET [38] and that reduced CSF Aβ might relate more to early-stage AD, whereas the amyloid load assessed by PET is indicative of disease progression [39].

Schreiber et al. demonstrated that baseline florbetapir PET, rated either visually or using a cerebellar SUVr, was predictive of conversion to AD in a large longitudinal cohort [24]. The prognostic value of the baseline PET profile with respect to subsequent cognitive evolution was also highlighted, consistent with prior results derived from a retrospective study [22]. Yet, the exact added diagnostic and prognostic value of amyloid PET semi-quantitative indices compared with CSF markers is still unclear, and, relatedly, the optimal reference region for SUVr computation remains to be defined. In the present study, we systematically compared baseline CSF markers and PET semi-quantitative indices in terms of diagnostic value regarding baseline cognitive status, as well as prognostic value in patients with MCI regarding cognitive decline and conversion to AD. In addition, we evaluated the performance of the SUVr computed using various well-established subcortical reference regions.

## Methods

### Subjects

In this study, we used participant data from the Alzheimer’s Disease Neuroimaging Initiative (ADNI), a multicentre project with approximately 50 medical centres and university sites across the United States and Canada [40]. The ADNI was launched in 2003 as a public-private partnership led by Principal Investigator Michael W. Weiner, MD. Its primary goal was to examine how brain imaging and other biomarkers can be used to measure the progression of MCI and early AD. Determination of sensitive and specific markers of very early AD progression is expected to help researchers and clinicians develop new treatments and monitor their effectiveness, as well as lessen the time and cost of clinical trials. A detailed description of the inclusion criteria can be found on the ADNI webpage (http://www.adni-info.org). Subjects were between 55 and 90 years old and willing and able to undergo all test procedures, including neuroimaging, and had agreed to undergo longitudinal follow-up.

Cognitively normal participants were the control subjects in the ADNI study. They showed no signs of depression, MCI or dementia. Participants with significant memory complaint (SMC) scored within the normal range for cognition but indicated concerns and exhibited slight forgetfulness. Early and late MCI participants reported an SMC either autonomously or via an informant or clinician. However, other cognitive domains showed no significant impairment, activities of daily living were preserved, and there were no signs of dementia. Participants with AD met the National Institute of Neurological and Communicative Disorders and Stroke/Alzheimer’s Disease and Related Disorders Association criteria for probable AD [41, 42].

Data were downloaded from the ADNI database (adni.loni.usc.edu) and included all subjects recruited in the ADNI-2 with complete available baseline data regarding cognitive assessment, CSF markers and PET Aβ quantitation. Our cross-sectional sample was made up of 677 subjects (157 control subjects, 95 with SMC, 301 with MCI among whom 153 had early MCI and 148 had late MCI, and 124 with AD at the time of the florbetapir scan; *see* Table 1) who were recruited between January 2011 and September 2013, and each had a baseline CSF collection and florbetapir session. The time delay between the lumbar puncture and the florbetapir PET was 11 ± 18 days. Our longitudinal sample was made up of the 396 subjects with SMC and MCI from the cross-sectional sample who had undergone an average clinical follow-up of 30 ± 16 months (*see* Table 2). Baseline visit and follow-up visits at 3, 6 and 12 months, then yearly, included complete cognitive assessment using the Geriatric Depression Scale, Mini Mental State Examination (MMSE), Clinical Dementia Rating (CDR) and Alzheimer’s Disease Assessment Scale–Cognitive Subscale (ADAS-cog). Diagnostic status and cognitive scores were extracted from the latest available dataset (‘DXSUM_PDXCONV_ADNIALL.csv’). For each participant in the longitudinal cohort, the mean annual change in cognitive scores was computed by taking the difference between the last cognitive evaluation and the baseline one and dividing by the time range. The last known diagnostic status was the one mentioned at the time of the last visit listed in the dataset. For each participant of the longitudinal cohort for whom the last status was AD, time to conversion was computed as the delay between the baseline visit and the first visit mentioning an AD status.

**Table 1:** Baseline demographics, apolipoprotein E status and cerebrospinal fluid markers in the cross-sectional population by baseline status

Baseline status	Control subjects (*n* = 157)	SMC/MCI (*n* = 396)	AD (*n* = 124)
Male sex	77 (49%)	204 (52%)	72 (58%)
Age, years	74 ± 6	72 ± 7^a^	75 ± 8
ApoE4 carriers	42 (27%)	186 (47%)^b^	82 (66%)^c^
Baseline cognition			
GDS	0.7 ± 1.1	1.4 ± 1.4^b^	1.7 ± 14^c^
MMSE	29 ± 1	27 ± 3^b^	23 ± 2^c^
CDR 0	157 (100%)	96 (24%)^b^	0^c^
CDR 0.5	0	299 (76%)^a^	53 (43%)^c^
CDR ≥1	0	1 (0.3%)	71 (57%)^c^
ADAS-cog	9 ± 5	16 ± 10^b^	31 ± 8^c^
Baseline CSF			
Aβ_1–42_, ng/L	196 ± 50	175 ± 53^b^	137 ± 38^c^
Tau, ng/L	67 ± 34	88 ± 54^b^	133 ± 65^c^
p-Tau_181_, ng/L	33 ± 16	43 ± 26^b^	60 ± 35^c^
p-Tau_181_/Aβ_1–42_	0.19 ± 0.13	0.29 ± 0.24^b^	0.48 ± 0.32^c^

*Abbreviations: Aβ* Amyloid-β_1–42_, *AD* Alzheimer’s disease, *ADAS-cog* Alzheimer’s Disease Assessment Scale–Cognitive Subscale, *ApoE* Apolipoprotein E, *CDR* Clinical Dementia Rating, *GDS* Geriatric Depression Scale, *MCI* Mild cognitive impairment, *MMSE* Mini Mental State Examination, *p-tau* Phosphorylated tau, *SMC* Significant memory complaint

^a^ *p* < 0.01 vs control subjects

^b^ *p* < 0.001 vs control subjects

^c^ *p* < 0.001 vs patients with SMC/MCI

**Table 2:** Baseline demographics, apolipoprotein E status, cerebrospinal fluid markers and clinical score evolution in the longitudinal cohort (patients with significant memory complaint/mild cognitive impairment) by last known status

Last known status	Normal (*n* = 105 [27%])	MCI (*n* = 209 [53%])	AD (*n* = 82 [21%])
Follow-up duration, months	25 ± 12	34 ± 16^a^	36 ± 13
Male sex	40 (38%)	121 (58%)^a^	43 (52%)
Age, years	71 ± 6	72 ± 7	73 ± 7
ApoE4 carriers	41 (39%)	87 (42%)	58 (71%)^b^
Baseline cognition			
GDS	1.1 ± 1.1	1.8 ± 1.4^a^	1.8 ± 14
MMSE	29 ± 1	28 ± 2^a^	27 ± 2^b^
CDR 0	86 (82%)	9 (4%)^a^	1 (1%)^b^
CDR 0.5	19 (18%)	199 (95%)^a^	81 (99%)^b^
CDR ≥1	0	1 (0.5%)	0
ADAS-cog	9 ± 4	14 ± 6^a^	22 ± 7^b^
Baseline CSF			
Aβ_1–42_ (ng/L)	204 ± 48	179 ± 53^a^	141 ± 35^b^
Tau (ng/L)	62 ± 30	77 ± 44^a^	121 ± 60^b^
p-Tau_181_ (ng/L)	35 ± 20	38 ± 23	59 ± 25^b^
p-Tau_181_/Aβ_1–42_	0.19 ± 0.15	0.25 ± 0.20^c^	0.44 ± 0.21^b^
Follow-up			
MMSE annual change	−0.1 ± 0.2	−0.4 ± 1.1	−2 ± 2.3^b^
CDR annual change	0.01 ± 0.14	0.00 ± 0.10	0.23 ± 0.21^b^
ADAS-cog annual change	−0.1 ± 2.3	0.5 ± 3.3	3.7 ± 3.8^b^
Time to conversion, months	–	–	22 ± 13

*Abbreviations: Aβ* Amyloid-β_1–42_, *AD* Alzheimer’s disease, *ADAS-cog* Alzheimer’s Disease Assessment Scale–Cognitive Subscale, *ApoE* Apolipoprotein E, *CDR* Clinical Dementia Rating, *GDS* Geriatric Depression Scale, *MCI* Mild cognitive impairment, *MMSE* Mini Mental State Examination, *p-tau* Phosphorylated tau, *SMC* Significant memory complaint

^a^ *p* < 0.001 vs normal patients

^b^ *p* < 0.001 vs patients with MCI

^c^ *p* < 0.01 vs normal patients

### CSF markers

Baseline Aβ_1–42_, total tau and phosphorylated p-tau_181_ (p-tau) were measured using the multiplex xMAP Luminex platform (Luminex Corp., Austin, TX, USA) with the INNO-BIA AlzBio3 kit (Innogenetics, Ghent, Belgium) [5, 43]. For this study, we used the archived dataset ‘UPENNBIOMK_MASTER.csv’. When multiple baseline CSF marker dosages were available, the median value was retained for subsequent analyses. The studied variables of CSF biomarker were Aβ, tau, p-tau and the p-tau/Aβ ratio. Additional analysis details and quality control procedures appear on the ADNI website.

### Amyloid PET data

Baseline Aβ deposition was visualised using ^18^F-florbetapir PET. Semi-quantitative PET results were retrieved from the latest available dataset (‘UCBERKELEYAV45_10_17_16.csv’). The methods for PET acquisition and analysis are described in more detail elsewhere [22, 44]. Florbetapir images consisted of 4 × 5-minute frames acquired at 50–70 minutes after injection, which were realigned, averaged, resliced to a common voxel size (1.5 mm) and smoothed to a common resolution of 8 mm in full width at half-maximum [45]. Structural T1-weighted images acquired concurrently with the baseline florbetapir images were used as a structural template to define the cortical regions of interest and the reference regions in native space for each subject, using FreeSurfer (version 4.5.0; surfer.nmr.mgh.harvard.edu) as described elsewhere [44]. Baseline florbetapir scans for each subject were co-registered to baseline structural magnetic resonance imaging scans, which were subsequently used to extract weighted cortical retention indices (SUV) from grey matter within four large cortical regions of interest (frontal, cingulate, parietal and temporal cortices) that were averaged to create a mean cortical SUV as described in greater detail online (adni.bitbucket.org/docs/UCBERKELEYAV45/UCBERKELEY_AV45_Methods_12.03.15.pdf). Cortical SUVr values were obtained by normalising cortical SUV with the mean uptake in a subcortical reference region. For the present study, candidate reference regions were pons, whole cerebellum and a composite region made up of the whole cerebellum, pons and eroded subcortical white matter [19]. In the sequel, the corresponding SUVr will be respectively referred to as pontine SUVr, cerebellar SUVr and composite SUVr.

### Statistical analyses

Continuous variables are presented as mean ± SD and categorical variables as number (percent). The diagnostic performance of CSF markers and SUVr was assessed through ROC analysis. For each parameter and each cut-off value, sensitivity was defined as the positivity rate in the patients with AD and specificity as the negativity rate in the control subjects/normal patients. The optimal cut-off value was that maximising Youden’s index (sensitivity + specificity − 1). The concordance between PET profile based on SUVr values and CSF profile was evaluated using Cohen’s kappa coefficient.

To test the association of baseline SUVr and CSF markers with diagnosis and prognosis, a multivariate analysis was conducted using a stepwise linear regression model with an entry criterion of *p* < 0.05 and a removal criterion of *p* > 0.1. To identify the independent determinants of baseline status and baseline cognition (MMSE, CDR and ADAS-cog), the following explicative factors were included in the model: sex, age, ApoE4 status, the four CSF variables and SUVr. To identify the independent predictors of final status, cognitive decline (annual change in MMSE, CDR and ADAS-cog) and time to conversion, the following explicative factors were included in the model: sex, age, ApoE4 status, baseline cognitive scores, the four CSF variables and SUVr. Categorical variables (sex, ApoE4 status, baseline and final status) were discretised, whereas (pseudo-)continuous variables (age, cognitive scores, CSF markers and SUVr) were processed as such. In each model, the three SUVr values based on the three candidate reference regions were tested separately, then jointly.

The correlation between baseline SUVr and cognitive score evolution was evaluated using least-squares quadratic regression and Spearman’s rank correlation. The statistical significance of the mean annual changes in cognitive scores was tested using a z-test.

The predictive value of baseline PET and CSF profiles regarding conversion to AD was assessed using Kaplan-Meier survival curves and the log-rank test. HRs were adjusted using a Cox proportional hazards model including the following explanatory covariates: sex, age, ApoE4 status, baseline cognitive scores, PET profile, CSF Aβ and tau profiles. For patients who did not convert to AD, survival data were considered censored from the time of the last visit on record.

A two-sided *p* value ≤0.05 was considered statistically significant. As regards the multivariate analysis, *p* values were corrected for multiple comparisons using the Dunn-Šidák correction: *p*_corrected_ = 1 − (1 − *p*)^*m*^, with *m* being the number of comparisons (here we set *m* = 9 as the number of times the linear model was run). All statistical computations were performed using MATLAB R2013 software (MathWorks, Natick, MA, USA).

## Results

**Fig. 1:**
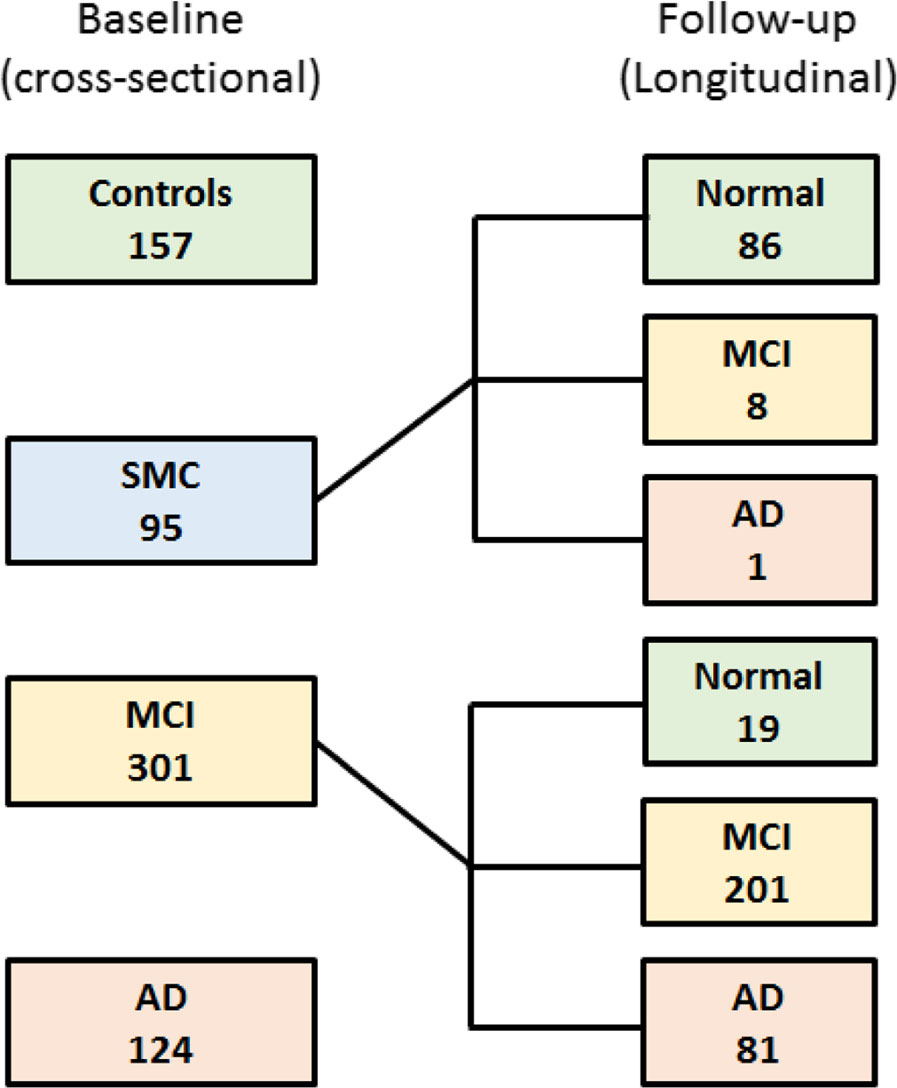
Patient flow diagram in the cross-sectional and longitudinal cohorts. *AD* Alzheimer’s disease, *MCI* Mild cognitive impairment, *SMC* Significant memory complaint

Figure 1 presents the patient flow diagram. For the cross-sectional cohort, the patient demographics, ApoE4 status, baseline cognitive scores and CSF markers are detailed in Table 1. The differences between control subjects and patients with MCI and between patients with MCI and patients with AD were highly significant for ApoE4 status, cognition and all four CSF markers. For the longitudinal cohort, the patient demographics, ApoE4 status, baseline cognition and CSF, annual change in cognitive scores during follow-up and time to conversion are detailed in Table 2. Of the 396 patients with SMC/MCI at baseline, 209 (53%) were classified as having MCI at their last visit, 105 (27%) were ranked as normal (mostly patients with baseline SMC, and 19 patients with baseline MCI who reverted to normal) and 82 (21%) converted to AD (1 SMC, 19 early MCI and 62 late MCI). The differences in baseline cognition and CSF markers were highly significant between normal subjects and patients with MCI and between patients with MCI and patients with AD. Cognitive decline was similar in normal subjects and patients with MCI and markedly greater in patients with AD.

**Fig. 2:**
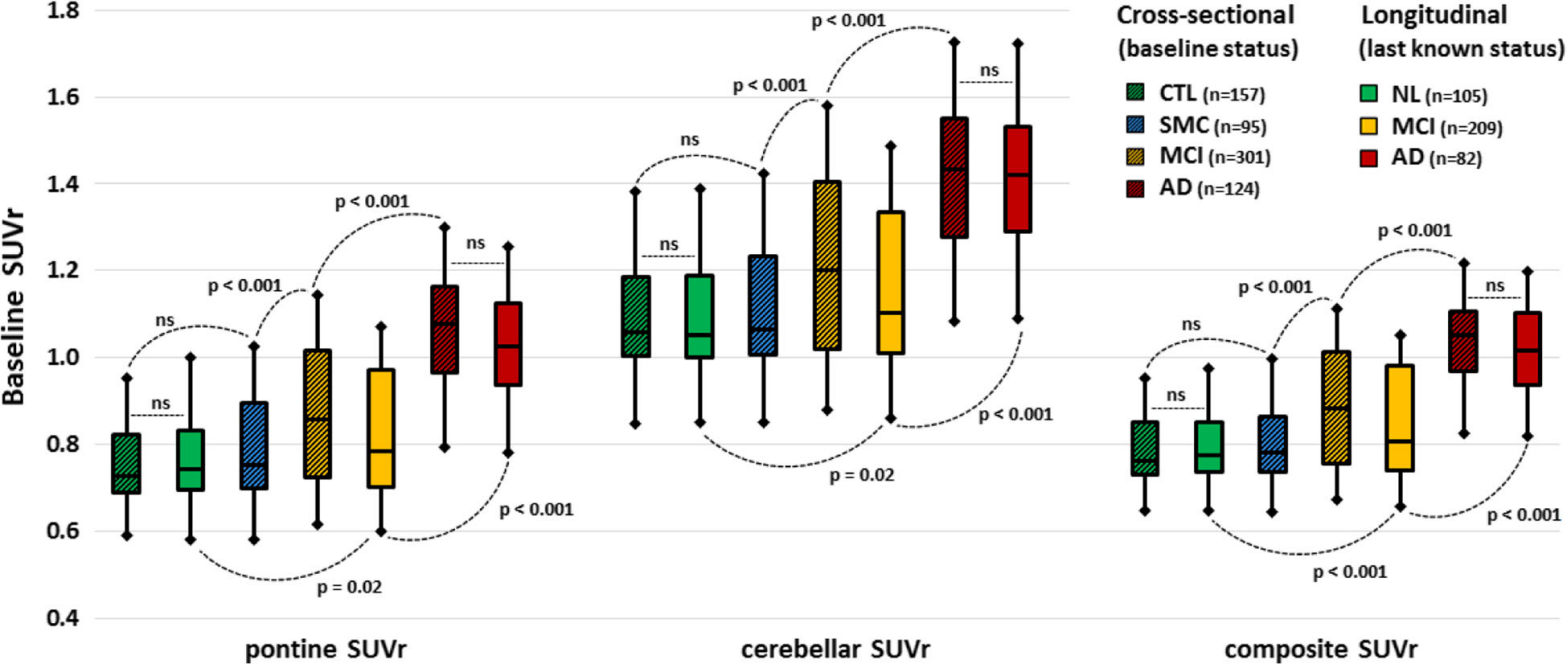
Distribution of baseline standardised uptake value ratio (SUVr) values by baseline status (control subjects, significant memory complaint [SMC], mild cognitive impairment [MCI], Alzheimer’s disease [AD]) in the cross-sectional cohort and by last known status (normal, MCI, AD) in the longitudinal cohort. Boxes represent IQRs. Whiskers correspond to mean ± 1.5 SD. *CTL* Control, *NL* Normal, *ns* Not significant

Figure 2 shows the distribution of (from left to right) pontine, cerebellar and composite SUVr values in the cross-sectional and longitudinal cohorts. In both cohorts, SUVr values were significantly lower in normal patients than in patients with MCI and in patients with MCI than in patients with AD, whatever the reference region used. No difference was found between the homologous subsets of normal and patients with AD from the two cohorts.

**Fig. 3:**
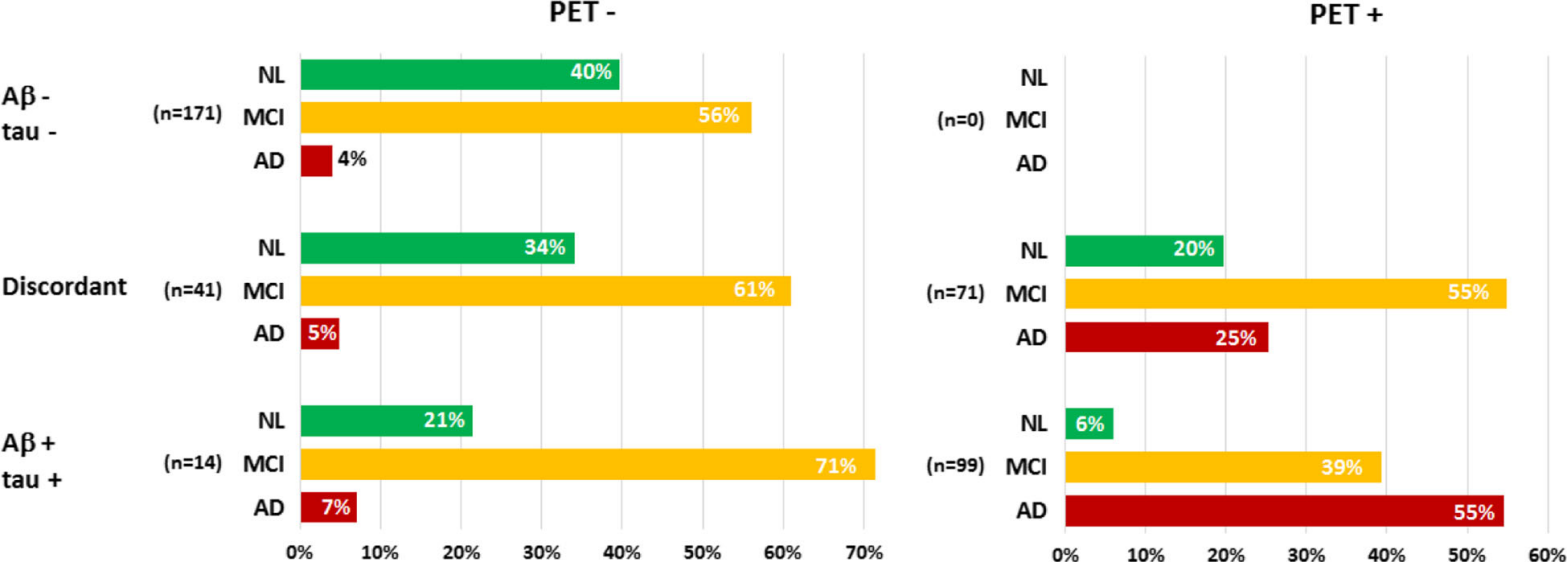
Last known status in the longitudinal cohort (patients with significant memory complaint/patients with mild cognitive impairment [MCI]) according to baseline positron emission tomography (PET) profile (assessed using composite standardised uptake value ratio with a cut-off at 0.89) and cerebrospinal fluid profile in terms of amyloid-β_1–42_ (Aβ/tau) combination. *AD* Alzheimer’s disease, *NL* Normal

Table 3 details the results of the ROC analyses for SUVr and CSF markers. Sensitivity and specificity stand for, respectively, the rate of true-positives among patients with AD and the rate of true-negatives among control subjects/normal patients. Optimal cut-off values for SUVr were highly similar in the cross-sectional and longitudinal cohorts, whereas they differed substantially for the CSF markers. SUVr performances were globally higher than those of CSF markers. In both cohorts, the best diagnostic performance was achieved using composite SUVr with an AUROC above 0.85, a sensitivity above 85% and a specificity above 80% in cross-sectional and longitudinal analyses. Overall, the predictive power of SUVr was superior to that of CSF markers, with risk ratios for evolving to AD ranging from 7 to 9.5 (vs 4.5 to 8 for CSF markers). Figure 3 shows the frequencies of final status in the longitudinal cohort according to baseline PET (composite SUVr) and baseline CSF profile (Aβ/tau combination). Seventy-two percent of the patients had concordant Aβ/tau profiles (43% negative, 29% positive), and 28% had discordant Aβ/tau profiles (25% Aβ^+^/tau^−^ and 3% Aβ^−^/tau^+^). There was no significant difference in mean follow-up duration between negative and positive profiles (PET, Aβ or tau).

**Table 3:** Results of ROC analyses for standardised uptake value ratio and cerebrospinal fluid markers

	SUVr (pons)	SUVr (crb)	SUVr (comp)	Aβ_1–42_	Tau	p-Tau_181_	p-Tau/Aβ
Cross-sectional cohort							
Optimal cut-off	0.91	1.22	0.91	157	69	32	0.18
AUC	0.88	0.84	0.88	0.83	0.85	0.79	0.85
Acc	85%	81%	85%	80%	75%	70%	77%
Se	85%	83%	87%	87%	90%	85%	93%
Sp	85%	79%	83%	75%	63%	59%	65%
Longitudinal cohort							
Optimal cut-off	0.91	1.24	0.89	171	88	45	0.27
AUC	0.85	0.84	0.86	0.85	0.81	0.80	0.85
Acc	82%	82%	84%	79%	79%	75%	82%
Se	83%	84%	88%	90%	68%	71%	83%
Sp	82%	81%	81%	70%	87%	79%	81%
PPV	43%	44%	42%	35%	44%	43%	44%
NPV	94%	95%	96%	96%	90%	91%	94%
RR	7.2	8.1	9.6	8.0	4.6	4.6	7.6

*Abbreviations: Acc* Accuracy, *Se* Sensitivity (positivity rate among patients with Alzheimer’s disease), *Sp* Specificity (negativity rate among control subjects/normal patients), *PPV* Positive predictive value, *NPV* Negative predictive value, *RR* Risk ratio for evolving to Alzheimer’s disease, *Pons* Pontine, *crb* Cerebellar, *comp* Composite

The concordance between the PET and CSF profiles was good when SUVr was compared with Aβ (kappa > 0.8) and moderate when it was compared with tau and p-tau (kappa around 0.6–0.7), without substantial variation related to the chosen reference region (*see* Table 4 for details).

**Table 4:** Concordance (kappa) between positron emission tomography and cerebrospinal fluid profiles in the cross-sectional and longitudinal cohorts

	Aβ_1–42_	Tau	p-Tau_181_	p-Tau/Aβ
Cross-sectional cohort				
SUVr (pons)	0.82	0.60	0.57	0.74
SUVr (crb)	0.81	0.60	0.60	0.75
SUVr (comp)	0.84	0.61	0.59	0.75
Longitudinal cohort				
SUVr (pons)	0.80	0.67	0.70	0.79
SUVr (crb)	0.78	0.65	0.69	0.81
SUVr (comp)	0.83	0.67	0.69	0.81

*Abbreviations: Aβ* Amyloid-β_1–42_, *p-tau* Phosphorylated tau, *SUVr* Standardised uptake value ratio, *Pons* Pontine, *crb* Cerebellar, *comp* Composite

Tables 5 and 6 summarise the results of the multivariate analyses. SUVr *p* values reported in the tables are those obtained when the three SUVr values were evaluated separately. An asterisk designates the *p* values that remained significant when the three SUVr values were evaluated jointly. The coefficients of determination (*r* ^2^) reflect the proportion of the variance in the modelled variable that is predictable from each explanatory variable retained in the model. Regarding the cross-sectional cohort (Table 5), sex, tau level and SUVr were independent determinants of baseline status and cognitive scores (all corrected *p* values <0.001), whereas ApoE4 status and other CSF variables were not. The best determinants were pontine and composite SUVr, which showed similarly high association with patient status and cognitive level. For the longitudinal cohort (Table 6), baseline cognition (MMSE, CDR and ADAS-cog) was the main predictor of cognitive decline in terms of final status and annual deterioration in cognitive scores. In patients with MCI who converted to AD during follow-up (*n* = 82), baseline ADAS-cog score was the sole independent predictor of time to conversion (*p* = 0.03). CSF markers showed little or no association with cognitive evolution (p-tau on final status *p* = 0.02, tau on MMSE change *p* = 0.02). On the contrary, pontine and composite SUVr yielded significant additional prognostic information about final status (*p* < 0.001) and cognitive score decline (*p* < 10^−5^ for CDR change, *p* = 0.001–0.005 for ADAS-cog change).

**Table 5:** Results of the stepwise linear regression investigating the association of baseline demographics, cerebrospinal fluid markers and positron emission tomography data, with baseline status and cognitive scores in the cross-sectional cohort

Association with	Baseline status (CTL, SMC/MCI, AD)		Baseline MMSE		Baseline CDR		Baseline ADAS-cog	
	*r* ^2^	*p* Value	*r* ^2^	*p* Value	*r* ^2^	*p* Value	*r* ^2^	*p* Value
Sex	0.04	<0.001	0.04	<10^−4^	0.04	<0.001	0.08	<10^−8^
Age		ns	0.03	0.003		ns	0.04	<0.001
ApoE4		ns		ns		ns		ns
Baseline CSF								
Aβ_1–42_		ns		ns		ns		ns
Tau	0.06	<10^−5^	0.10	<10^−9^	0.07	<10^−7^	0.12	<10^−11^
p-Tau_181_		ns		ns		ns		ns
p-Tau/Aβ		ns		ns		ns		ns
Baseline PET								
SUVr (pons)	0.18	<10^−19(*)^	0.14	<10^−15^	0.14	<10^−15^	0.18	<10^−24^
SUVr (crb)	0.10	<10^−6(*)^	0.02	0.03 ^(*)^		ns	0.05	0.001 ^(*)^
SUVr (comp)	0.17	<10^−17^	0.14	<10^−14(*)^	0.14	<10^−14(*)^	0.19	<10^−23(*)^

*Abbreviations: AD* Alzheimer’s disease, *ADAS-cog* Alzheimer’s Disease Assessment Scale–Cognitive Subscale, *ApoE* Apolipoprotein E, *CDR* Clinical Dementia Rating, *Comp* Composite, *Crb* Cerebellar, *CSF* Cerebrospinal fluid, *MCI* Mild cognitive impairment, *MMSE* Mini Mental State Examination, *Pons* Pontine, *SMC* Significant memory complaint, *SUVr* Standardised uptake value ratio, *ns* Not significant

All *p* values are corrected for multiple comparisons

(*): Remained an independent determinant when evaluated jointly

**Table 6:** Results of the stepwise linear regression investigating the association of baseline demographics, cognitive scores, cerebrospinal fluid markers and positron emission tomography data, with prognosis in terms of final status, cognitive score evolution and time to conversion in the longitudinal cohort

Association with	Last known status (NL, MCI, AD)		MMS annual change		CDR annual change		ADAS-cog annual change		Time to conversion	
	*r* ^2^	*p* Value	*r* ^2^	*p* Value	*r* ^2^	*p* Value	*r* ^2^	*p* Value	*r* ^2^	*p* Value
Sex		ns		ns		ns		ns		ns
Age		ns		ns		ns		ns		ns
ApoE4		ns		ns		ns		ns		ns
Baseline cognition										
GDS		ns		ns	0.02	0.04		ns		ns
MMSE		ns	0.10	<10^−8^		ns	0.10	<0.001		ns
CDR	0.38	<10^−16^		ns	0.08	<10^−8^		ns		ns
ADAS-cog	0.18	<10^−15^	0.18	<10^−18^	0.14	<10^−16^		ns	0.10	0.03
Baseline CSF										
Aβ_1–42_		ns		ns		ns		ns		ns
Tau		ns	0.03	0.02		ns		ns		ns
p-Tau_181_	0.03	0.02		ns		ns		ns		ns
p-Tau/Aβ		ns		ns		ns		ns		ns
Baseline PET										
SUVr (pons)	0.05	<0.001^a^		ns	0.06	<10^−5a^	0.08	0.001^a^		ns
SUVr (crb)	0.03	0.02		ns		ns		ns		ns
SUVr (comp)	0.05	<0.001		ns	0.05	<10^−5^	0.07	0.005		ns

*Abbreviations: Aβ* Amyloid-β_1–42_, *AD* Alzheimer’s disease, *ADAS-cog* Alzheimer’s Disease Assessment Scale–Cognitive Subscale, *ApoE* Apolipoprotein E, *CDR* Clinical Dementia Rating, *Comp* Composite, *Crb* Cerebellar, *CSF* Cerebrospinal fluid, *GDS* Geriatric Depression Scale, *MCI* Mild cognitive impairment, *MMSE* Mini Mental State Examination, *NL* Normal, *PET* Positron emission tomography, *Pons* Pontine, *p-tau* Phosphorylated tau, *SUVr* Standardised uptake value ratio, *ns* Not significant

All *p* values are corrected for multiple comparisons

^a^Remained an independent predictor when evaluated jointly

**Fig. 4:**
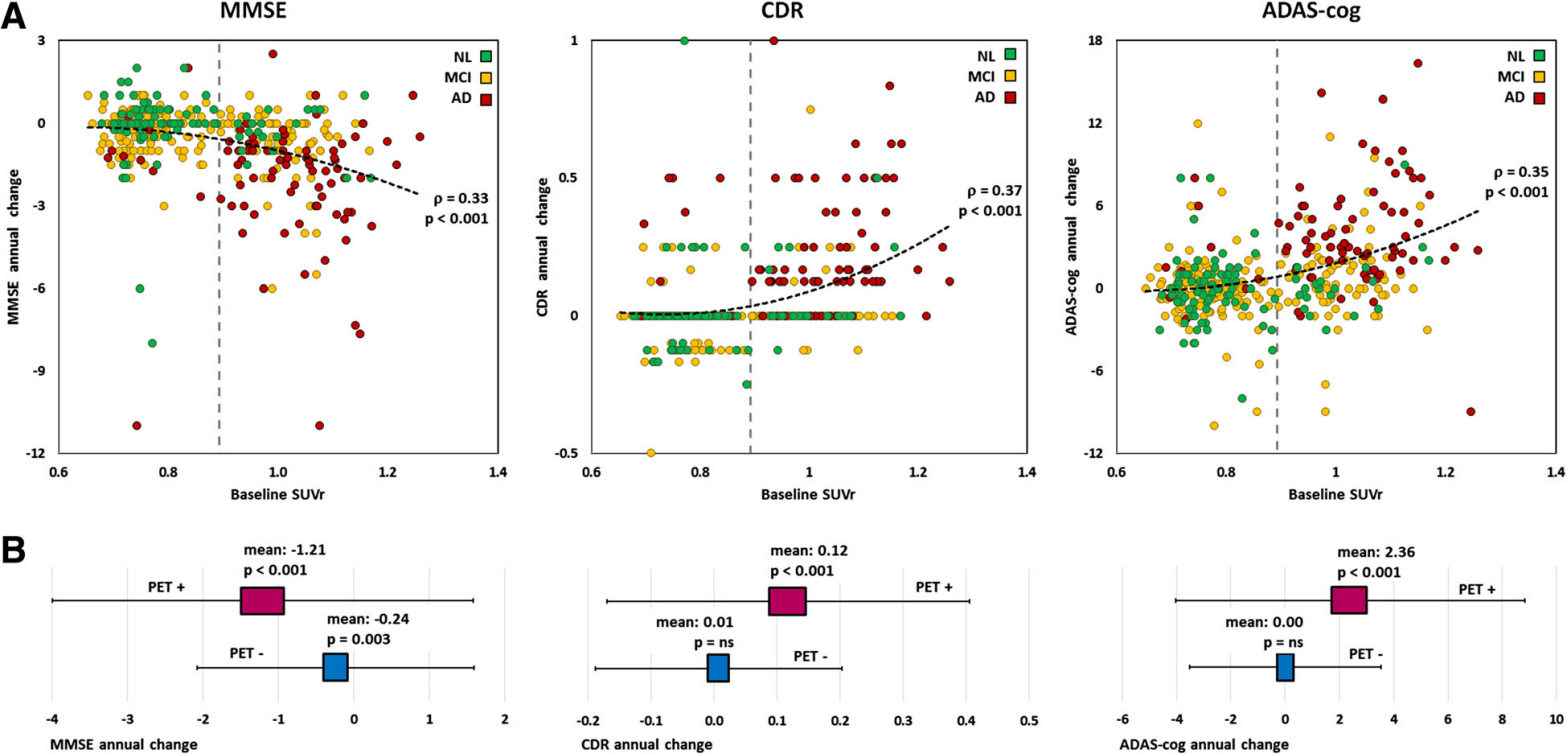
Evolution of cognitive scores in the longitudinal cohort (patients with significant memory complaint/mild cognitive impairment [MCI]) according to baseline composite standardised uptake value ratio (SUVr). *From left to right*: Mean annual change in Mini Mental State Examination (MMSE), Clinical Dementia Rating (CDR) and Alzheimer’s Disease Assessment Scale–Cognitive Subscale (ADAS-cog). **a** Scatterplot in which marker colour refers to last known status. The *vertical dashed grey line* indicates the optimal SUVr cut-off. The *black dashed curve* stands for the quadratic regression. *ρ* Spearman’s rank correlation. **b** Score distribution according to positron emission tomography (PET) profile based on composite SUVr (cut-off 0.89). Boxes represent mean with 95% CI. Whiskers represent mean ± 1.5 SD. *ns* Not significant, *NL* Normal, *AD* Alzheimer’s disease

The prognostic relevance of SUVr is further elaborated in Fig. 4. The scatterplots of the annual change in cognitive scores are displayed on top (Fig. 4a) according to the baseline composite SUVr, as are the associated quadratic regressions based on the whole longitudinal cohort. There was a moderate but significant Spearman’s correlation of composite SUVr with cognitive decline (*r* = 0.33–0.37, all *p* values <0.001). The range and mean value (along with the 95% CI) of the score changes according to the baseline PET profile are shown in Fig. 4b. Patients with negative baseline PET incurred no significant change in CDR and ADAS-cog during follow-up. Conversely, patients with positive baseline PET exhibited clinically and statistically significant cognitive decline with mean annual changes of −1.2 in MMSE, +0.12 in CDR and +2.4 in ADAS-cog (all *p* values <0.001).

**Fig. 5:**
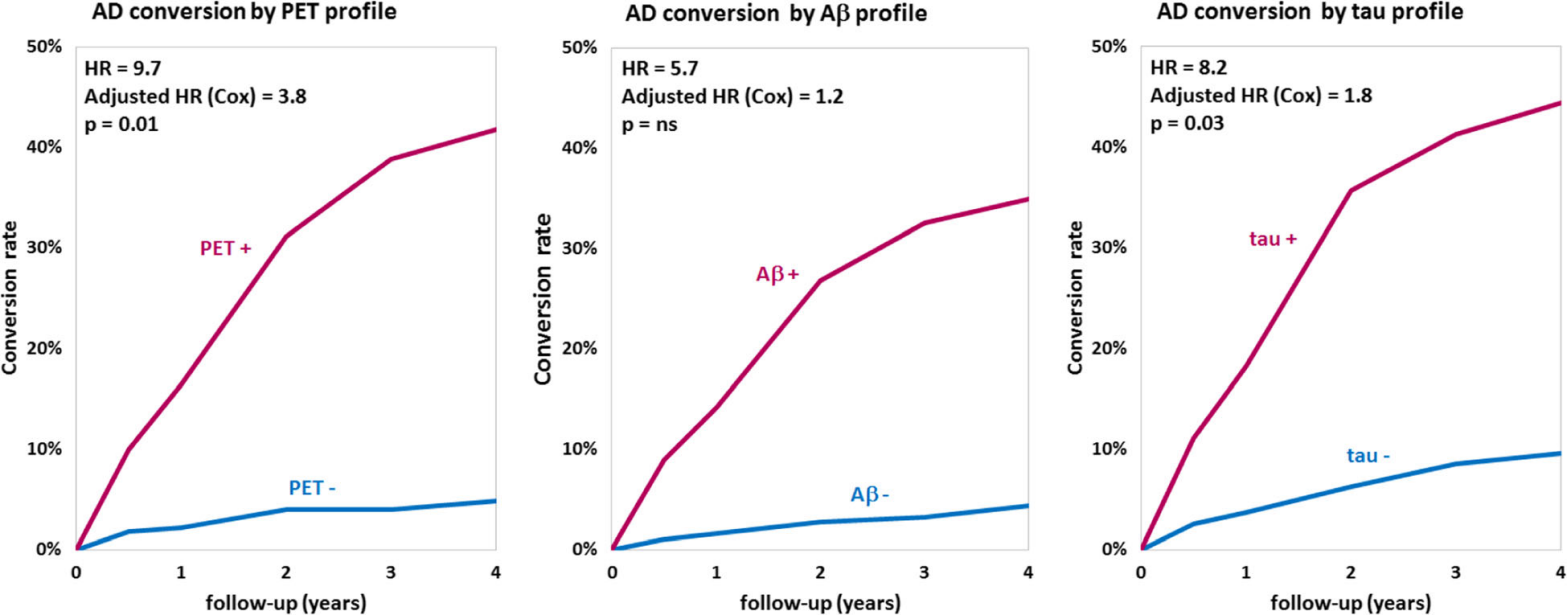
Kaplan-Meier curves for conversion to Alzheimer’s disease (AD) in patients with significant memory complaint/mild cognitive impairment (MCI) according to baseline positron emission tomography (PET) profile (composite standardised uptake value ratio [SUVr]) and baseline cerebrospinal fluid (CSF) Amyloid-β_1–42_ (Aβ) and total tau profiles. *ns* Not significant. Cox model accounts for age, sex, apolipoprotein E status, baseline cognitive scores, PET profile, CSF Aβ and tau profiles

Figure 5 presents the Kaplan-Meier curves for conversion to AD in patients with SMC/MCI according to baseline PET (composite SUVr) and CSF profiles. The Cox proportional hazards model shows that baseline ADAS-cog score was the strongest predictor for AD conversion (*p* < 10^−8^). A positive baseline PET was associated with an adjusted HR of 3.8 for AD conversion (*p* = 0.01). CSF Aβ and tau were less predictive with adjusted HRs of 1.2 (not significant) and 1.8 (*p* = 0.03), respectively.

## Discussion

In this study based on prospective data from the ADNI-2 cohort, we examined the complementary diagnostic and prognostic value of baseline CSF markers and ^18^F-florbetapir SUVr values computed using three different reference regions. We found that PET semi-quantitative assessment of Aβ load was significantly superior, although CSF and PET markers were both relevant determinants of cognitive status and predictive of cognition decline in patients with MCI. Notably, as can be seen in Fig. 2 and Table 3, baseline SUVr distribution was similar in patients with baseline AD and patients with SMC/MCI who converted to AD during follow-up; hence, the optimal SUVr cut-offs to differentiate patients with AD from normal subjects were nearly identical in the cross-sectional and longitudinal cohorts. The optimal cut-offs for CSF markers were less robust, suggesting that PET quantitation might be preferable for accurate selection and therapeutic monitoring of individuals in clinical trials [46].

Our optimal cerebellar SUVr cut-off (1.22) was consistent with that proposed by Fleisher et al. (1.17), based on post-mortem neuropathological data [47]. A less conservative SUVr cut-off was proposed by Joshi et al. [17] as the upper bound of a one-tailed 95% CI of cerebellar SUVr distribution in young healthy control subjects, and it was used in other studies [22, 24] as a positivity threshold for florbetapir PET. Such a low threshold based on young control subjects seems questionable, however, and may result in poor specificity (about 70% in the study by Landau et al. [22]), given that significant amyloid deposition without cognitive impairment is seen in 20% to 40% of normal elderly volunteers [14, 48]. To our knowledge, this is the first attempt to provide optimal thresholds for pontine and composite SUVr, because recent studies involving extra-cerebellar reference regions have been aimed primarily at assessing the longitudinal accuracy of SUVr estimates [18, 19].

Regarding the CSF ROC analyses, our optimal Aβ cut-off to differentiate patients with AD from normal control subjects (157 ng/L) was similar to that obtained by De Meyer et al. (159 ng/L) based on the ADNI-1 cohort [12]. Our optimal CSF Aβ cut-off to predict conversion to AD in the longitudinal analysis (171 ng/L) was closest to that proposed by Shaw et al. (192 ng/L) with reference to autopsy data [5], yielding comparable sensitivity and negative predictive value (respectively, 90% and 96% vs 96% and 95%). Our optimal cut-off for CSF tau (88 ng/L) was also similar to that mentioned by Shaw (93 ng/L) [5]. The proportion of concordant CSF profiles in terms of Aβ and tau was 72% in both cohorts, concordant with the 73% of concordant profiles reported by Sunderland et al. [4] in a cohort of patients with AD and control subjects.

In our cross-sectional cohort, the first interesting finding was that PET SUVr clearly outperformed CSF markers in determining patients’ cognitive status, as evaluated in a multivariate model. Its diagnostic accuracy neighboured 85% in differentiating patients with AD from control subjects, and cognitive performance (MMSE, CDR and ADAS-cog) was significantly associated with pontine and composite SUVr in the whole population. The higher diagnostic performance of pontine and composite SUVr than cerebellar SUVr might be related to a lower signal-to-noise ratio in the cerebellum, leading to less accurate and more variable SUV measurements in this region. Researchers in previous studies pointed out that pontine and cerebellar uptake was prone to noise and longitudinal variability owing to the small size of the considered regions and their peripheral location in the PET scanner field of view, and they advocated for the use of composite reference regions taking into account cerebral white matter [18, 19].

The CSF markers showed lower diagnostic value in ROC analysis (lower AUC and lower accuracy of 80% for Aβ and 75% for tau), and total tau was the sole CSF marker to bring added diagnostic value. Palmqvist et al. [35] noted that ^18^F-flutemetamol cerebellar SUVr was correlated with global cognition and hippocampal atrophy in patients with increased Aβ load, whereas CSF Aβ was not. These data are consistent with a commonly accepted model of AD pathological cascade, according to which Aβ deposition takes place at an early stage in the natural history of the disease and tau-mediated neuronal injury occurs secondarily [3]. Yet, although CSF Aβ reaches a plateau prior to the prodromal state, PET retention gradually increases during progression to AD [49]. Semi-quantitative amyloid PET may thus be more appropriate than CSF markers for early-stage grading of AD. To be fully operative and allow efficient discrimination between neurodegenerative diseases, it has to be integrated within the range of available biomarkers, including tau-specific PET tracers currently under clinical assessment [50].

The second original finding, which might have stronger practical implications, was that baseline PET SUVr was more predictive of clinical evolution and AD conversion than CSF markers and that baseline SUVr levels directly correlated with the subsequent rate of cognitive decline. Composite SUVr predictive accuracy regarding final status reached 84% compared with 79% for both CSF Aβ and tau. In line with prior reports, cognitive measures at baseline were the best predictors of cognitive evolution and AD conversion [51, 52]. Baseline pontine and composite SUVr were moderate but significant predictors of final status and mean annual CDR and ADAS-cog change in multivariate analysis, whereas CSF markers had little or no impact on cognitive evolution. Cognitive decline as reflected by the mean annual changes in MMSE, CDR and ADAS-cog was significantly correlated with baseline composite SUVr. The mean annual changes in CDR and ADAS-cog were significant in patients with positive baseline PET, whereas patients with negative baseline PET did not incur significant CDR and ADAS-cog modification during follow-up (Fig. 4). Among patients with a negative baseline PET (rated using composite SUVr), 4% were AD converters, and among those with a positive PET scan, 42% were AD converters. This yielded an adjusted HR for AD conversion of 3.8 (*p* = 0.01). Notably, the PET profile appeared decisive in patient with discordant CSF markers (99 Aβ^+^/tau^−^ and 13 Aβ^−^/tau^+^). In these patients, an abnormal amyloid PET resulted in a five-fold increase in AD conversion risk (25% vs 5% in patients with a normal amyloid PET; *see* Fig. 3). It would seem that even in patients with a concordant positive CSF profile (Aβ^+^/tau^+^), a negative PET is associated with a moderate risk of AD conversion (7% vs 55% in patients with a positive PET), though Aβ^+^/tau^+^/PET^−^ profiles were too few to ensure sufficient statistical power. Patients with a negative PET profile who evolved to AD during follow-up might either correspond to PET false-negatives or to cases of non-amyloid dementias. The proportion of PET-positive patients who were ranked as normal during follow-up is consistent with previous evidence that 20% to 30% of cognitively normal elderly subjects harbour Aβ deposition [53].

## Conclusions

Semi-quantitative amyloid PET and CSF markers yield complementary information for classifying normal subjects, patients with MCI and patients with AD. However, PET might be preferable for robust grading of early-stage AD, and cross-sectional cut-off values for SUVr seem to be directly transposable for longitudinal analysis. Amyloid PET quantification using a composite SUVr appears more powerful than CSF markers for MCI prognosis in terms of AD conversion, and progressive cognitive decline is correlated with baseline composite SUVr. In patients with an equivocal CSF profile, amyloid PET effectively differentiates patients with high risk of AD conversion.

## Abbreviations

Aβ: Amyloid-β_1–42_
Acc: Accuracy
AD: Alzheimer’s disease
ADAS-cog: Alzheimer’s Disease Assessment Scale–Cognitive Subscale
ADNI: Alzheimer’s Disease Neuroimaging Initiative
ApoE: Apolipoprotein E
CDR: Clinical Dementia Rating
Comp: Composite
Crb: Cerebellar
CSF: Cerebrospinal fluid
GDS: Geriatric Depression Scale
MCI: Mild cognitive impairment
MMSE: Mini Mental State Examination
NPV: Negative predictive value
PET: Positron emission tomography
PiB: Pittsburgh Compound B
Pons: Pontine
PPV: Positive predictive value
RR: Risk ratio
Se: Sensitivity
SMC: Significant memory complaint
Sp: Specificity
SUV: Standardised uptake value
SUVr: Standardised uptake value ratio
